# Supplementary material for: A Chemiluminescent Method for the Detection of H2O2 and Glucose Based on Intrinsic Peroxidase-Like Activity of WS2 Quantum Dots
Source: Molecules. 2019 Feb 14;24(4):689. doi: 10.3390/molecules24040689 (PMC6413195; doi:10.3390/molecules24040689)
Supplement: Supplementary file 1 [file molecules-24-00689-s001.pdf]

molecules

MDPI

Article

A Chemiluminescent Method for the Detection of H_2O_2 and Glucose Based on Intrinsic Peroxidase-Like Activity of WS_2 Quantum Dots

Mahsa Haddad Irani-nezhad, [1,2], Alireza Khataee,[2,3,*], Javad Hassanzadeh [2] and Yasin Orooji [1,*]

1. College of Materials Science and Engineering, Nanjing Forestry University, No. 159, Longpan Road, Nanjing 210037, Jiangsu, China; mah_t_haddad@yahoo.com
2. Research Laboratory of Advanced Water and Wastewater Treatment Processes, Department of Applied Chemistry, Faculty of Chemistry, University of Tabriz, Tabriz 51666-16471, Iran; javadhassanzadeh63@gmail.com
3. Health Promotion Research Center, Iran University of Medical Sciences, Tehran 1449614535, Iran

* Correspondences: a_khataee@tabrizu.ac.ir (A.K.); yasin@njfu.edu.cn (Y.O.)

Academic Editors: Bidisha Sengupta and Derek J. McPhee

Received: 8 January 2019; Accepted: 13 February 2019; Published: 14 February 2019

Abstract: Currently, researchers are looking for nanomaterials with peroxidase-like activity to replace natural peroxidase enzymes. For this purpose, WS_2 quantum dots (WS_2 QDs) were synthesized via a solvothermal method, which improved the mimetic behavior. The resulting WS_2 QDs with a size of 1–1.5 nm had a high fluorescence emission, dependent on the excitation wavelength. WS_2 QDs with uniform morphology showed a high catalytic effect in destroying H_2O_2. The peroxidase-like activity of synthesized nanostructures was studied in H_2O_2 chemical and electrochemical reduction systems. The mimetic effect of WS_2 QDs was also shown in an H_2O_2–rhodamine B (RB) chemiluminescence system. For this aim, a stopped-flow chemiluminescence (CL) detection system was applied. Also, in order to confirm the peroxidase-like effect of quantum dots, colorimetry and electrochemical techniques were used. In the enzymatic reaction of glucose, H_2O_2 is one of the products which can be determined. Under optimum conditions, H_2O_2 can be detected in the concentration range of 0–1000 nmol·L^{-1}, with a detection limit of 2.4 nmol·L^{-1}. Using this CL assay, a linear relationship was obtained between the intensity of the CL emission and glucose concentration in the range of 0.01–30 nmol·L^{-1}, with a limit of detection (3S) of 4.2 nmol·L^{-1}.

Keywords: nanosensor; WS_2 quantum dots; peroxidase-like activity; chemiluminescence; glucose detection

1. Introduction

Hydrogen peroxide, used in biological, pharmaceutical, clinical, food, chemical, and environmental processes, became an interesting subject for research since its discovery (1818). Hydrogen peroxide, as one of the most reactive oxygen forms, is an essential intermediate in different fields such as sterilization, paper bleaching, liquid fuel cells, and organic oxidation [1]. Many analytical methods were developed to measure hydrogen peroxide, namely fluorescence [2], colorimetric [3], resonance dispersion [4], chromatography [5], and electrochemical methods [6].

Glucose is a simple sugar that circulates in the blood of animals as blood glucose. Containing six carbon atoms, glucose is a subset of monosaccharides which is considered as a hexose. D-Glucose is one of 16 aldo-hexosuric stereoisomers. It can be obtained by hydrolyzing carbohydrates

such as lactose, sucrose, maltose, cellulose, glycogen, etc. A higher or lower concentration than 4.4–6.6 mmol·L^{-1} in human blood causes serious hyperglycemia and hypoglycemia, respectively [7].

Various analytical methods used to determine H_2O_2 and glucose are not suitable for fast and economical measurement. Some of these methods are simple and economical, but have low sensitivity; some are sensitive, selective and valid, whereas they are time-consuming, expensive, or need skilled personnel; and others are quick and sensitive, but they are not selective [3]. Meanwhile, chemiluminescence (CL) attracted a lot of attention due to its high sensitivity, simple arrangement, fast response, and low background signal. CL, as a phenomenon, occurs when the product in an excited electronic state, resulting from an exoergic reaction, returns to the ground state via the emission of photons [8]. CL assays are applied in batch, flow injection mode, or stopped-flow procedures [9,10]. Both flow and batch CL processes can forgather as stopped flow [11].

Pokropiony and Ivanovsky presented a classification for nanomaterials, which includes zero- (0D), one- (1D), two- (2D), and three-dimensional (3D) nanomaterials [12]. Zero-dimensional nanomaterials, including uniform particle arrangements (quantum dots), shell–cavity quantum dots, particle heterogeneous arrangements, layered nanostructures, nanolenses, and hollow cores were synthesized by various research groups [13–16]. The 0D nanoparticles, such as quantum dots (QDs), are widely used in light-emitting diodes (LEDs) [17], solar cells [18], single-electron transistors [19], and lasers [20]. The QDs are crystals in the nanometer range, so that hundreds to a few thousand atoms can fit in the same direction. They are composed of a semiconductor, such as silicon, and because they act like an atom, they are called synthetic atoms. The crystal lattice of semiconductor QDs affects the electronic wave function. The QDs have a special energy spectrum, depending on the band gap and the special density of the electron-out-of-crystal mode [21,22].

Peroxidases constitute a large family of enzymes that normally catalyze the reactions as follows:

$$ROOR' + 2e^- + 2H^+ \rightarrow ROH + R'OH\,.$$

Hydrogen peroxide and organic hydro-peroxides such as lipid peroxides are favorable substrates for peroxidase. The nature of the electron donor is highly dependent on the enzyme's structure. The peroxidase activity of enzymes has enormous practical applications, such as reducing toxicity by catalyzing the oxidation of organic materials, eliminating dyes in wastewater refinement, or use as a diagnostic tool [23]. Despite the limitations of the natural enzyme, such as enzyme structural changes by pH, temperature, and solvent variations, the short life span of the enzyme, and the cost-effective preparation and purification process, it is widely used in the manufacture of sensors for the detection of the oxidase products of enzymatic reactions [24,25]. Many artificial enzymes were synthesized to compensate for the limitations of natural enzymes and improve the stability of these catalysts over a wide range of pH and temperature [26]. Some nanomaterials including MoS_2 nanosheets [27], Co_3O_4 nanoparticles [28], V_2O_5 nanowires [29], carbon nanotubes [30], graphene oxide [31], and carbon QDs [32] have catalytic peroxidase activity.

Transition metal dichalcogenides are layered compounds with weak interlayer interactions and strong bindings within the layers. WS_2 is one of the members of this family that is applied as a lubricant, catalyst, a component of lithium batteries, and so on [33,34]. Nanoscale WS_2 has desirable properties such as high specific surface area and remarkable electronic properties that bulk WS_2 materials do not demonstrate [35,36]. There are several methods to synthesize WS_2 nanosheets such as liquid exfoliation [37–39], chemical vapor deposition [40], and mechanical exfoliation [41]. WS_2 QDs can be generated by reducing the lateral size of nanosheets [42–44].

In this study, WS_2 QDs were synthesized using a solvothermal process. The synthesized quantum dots were characterized using transmission electron microscopy (TEM), X-ray diffraction (XRD), and photoluminescence (PL) techniques. Then, the peroxidase-like activity of the synthesized quantum dots was studied in the chemical and electrochemical reduction of H_2O_2 systems. For this aim, CL, colorimetry, and electrochemical techniques were exploited. Effective parameters in selected systems, including type and concentration of reagents such as rhodamine B (RB), sodium dodecyl sulfate (SDS), and QDs, were optimized. Finally, the suggested method was

tested for determination of glucose in real samples. Glucose participates in an enzymatic oxidation reaction, leading to the production of hydrogen peroxide, which then can be detected by a developed CL system based on WS_2 QDs. The product of this reaction has a strong CL emission proportional to the H_2O_2 or glucose concentration (Scheme 1).

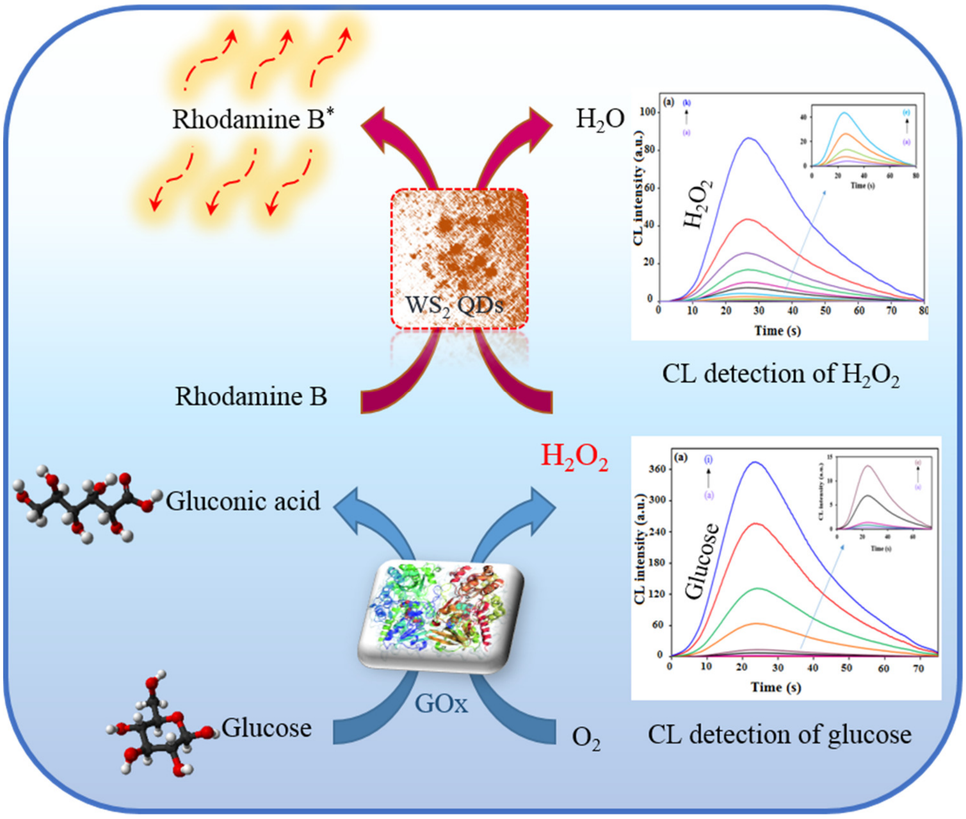

Scheme 1. Schematic design for the chemiluminescence (CL) determination of glucose based on the peroxidase-like activity of WS_2 quantum dots (QDs).

2. Results and Discussion

2.1. Study of Chemical Structure and Morphology of WS_2 QDs

In order to study the crystal phase and purity of the as-synthesized WS_2 QDs, XRD was utilized. As reported in previous literature, WS_2, in the form of QDs, does not show any signal in XRD pattern [37,45]. The results showed no peak that was related to the very small sizes of QDs (Figure S1, Supplementary Materials).

The morphology of WS_2 QDs was investigated by TEM and high-resolution TEM (HRTEM). As seen in Figure 1, QDs are high crystalline. Synthesized WS_2 nanocrystals showed an average diameter of 1.057 nm and a lattice spacing of 2.04 Å, which corresponds to the (006) phase of the WS_2 crystal (JCPDS 08-0237). The selected area electron diffraction pattern (SAED) was used for further verification of the structure. The results confirmed WS_2 QDs as being polycrystalline, distinct from bulk WS_2. This demonstrates that the bulk WS_2 was exfoliated during the sonication and hydrothermal processes.

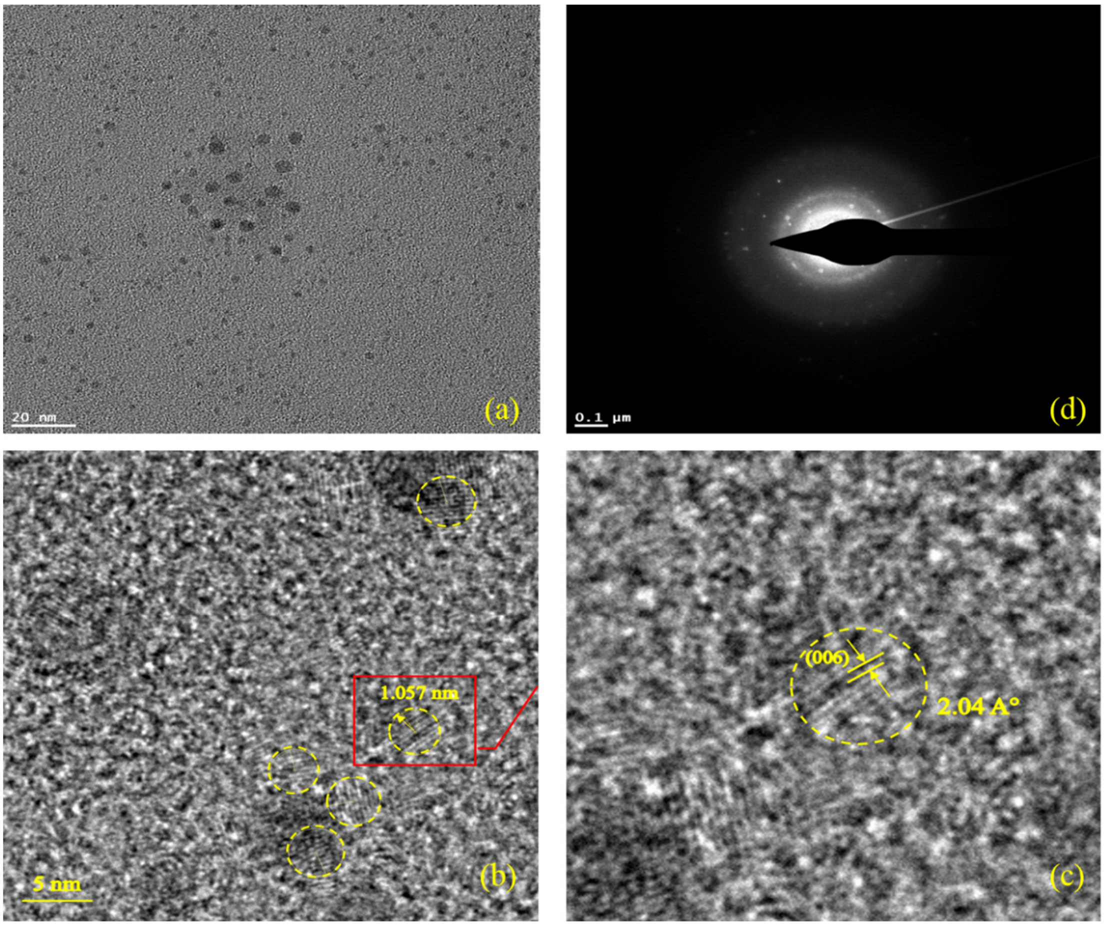

Figure 1. (**a**) TEM; (**b**,**c**) high-resolution TEM (HRTEM); and (**d**) selected area electron diffraction pattern (SAED) images for WS_2 quantum dots (QDs).

The solution of WS_2 QDs was clear and yellow and showed bright-blue PL. As shown in Figure 2a, only a weak peak around 300 nm was observed due to the excitonic feature of the QDs. Figure 2b indicates the PL spectra of the WS_2 QD solution prepared by the method described above at a distinct excitation wavelength. Changing the excitation wavelength in the range of 315–350 nm caused shifting of the fluorescence peak from 400 nm to 440 nm. It was observed that the maximum emission was at about 420 nm with an excitation wavelength of 330 nm. The intensity of the fluorescence increased up to λ_{ex} = 330 nm and then decreased, as observed in the previously reported WS_2 QDs synthesis methods [46,47]. Changing the electronic structure of WS_2 from an indirect band gap to a direct band gap led the observation of PL in QDs. The strong peak at 420 nm (with λ_{ex} = 330) was attributed to the strong quantum confinement with a shoulder at 435 nm (with λ_{ex} = 350) due to the incorporation of defect states created during exfoliation.

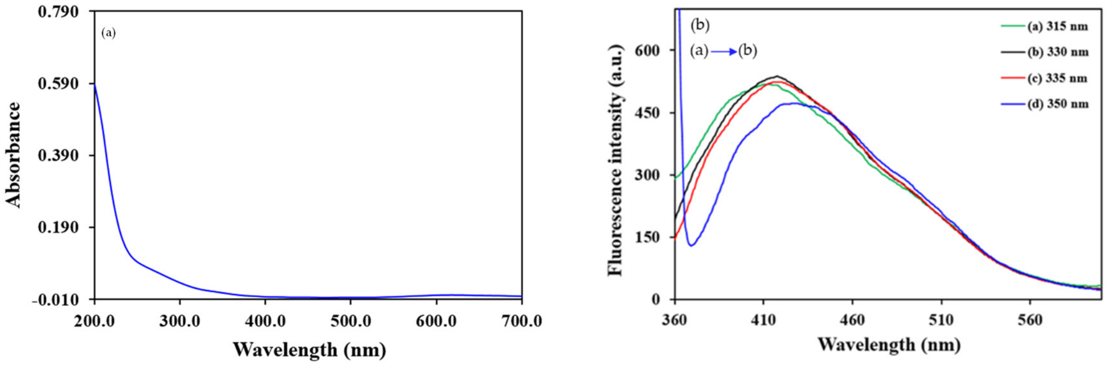

Figure 2. (**a**) Ultraviolet–visible (UV–Vis) absorption spectra of WS_2 QDs; (**b**) photoluminescence (PL) spectra of WS_2 QDs for different excitation wavelengths.

2.2. *Investigation of Peroxidase-Like Activity of WS_2 QDs*

Initial experiments demonstrated that WS_2 QDs have intrinsic peroxidase mimetic activity in the dissociation reaction of H_2O_2 to hydroxyl radicals, which offers many advantages in comparison with horseradish peroxidase (HRP). Several detection methods such as colorimetry, electrochemical methods, and CL were applied to investigate the peroxidase effect of synthesized QDs. It is worth mentioning that all experiments were done in optimized conditions (including type of buffer, pH, and concentration of reactants).

2.2.1. Colorimetry Investigations

The compound 3,3′,5,5′-tetramethylbenzidine (TMB) was used as a peroxidase substrate to study the peroxidase mimetic behavior of prepared QDs. WS_2 QDs can catalyze the oxidation of substrate by reacting with H_2O_2 in order to produce the colorful productions. As a result of TMB oxidation, a product with the maximum absorbance at the wavelength of 652 nm was generated (Figure 3). As can be seen from the results, the solution including WS_2 QDs was more colorful than the others. Moreover, in the absence of H_2O_2 or QDs, no reactions happened between TMB and H_2O_2, indicating that the presence of both is necessary for TMB oxidation, just as in the case occurring with natural peroxidase HRP.

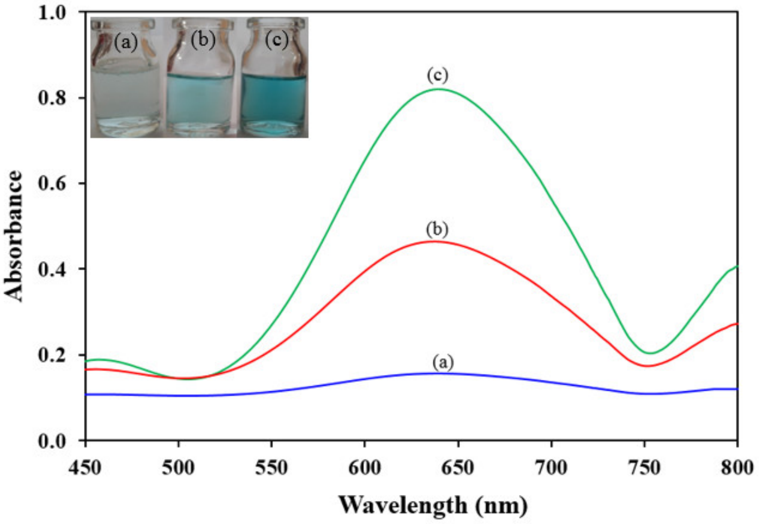

Figure 3. UV–Vis spectra of 3,3′,5,5′-tetramethylbenzidine (TMB; 4×10^{-1} mmol·L^{-1})–H_2O_2 solution in the (**a**) absence and (**b**) presence of HRP; and (**c**) WS2 QDs (1 mmol·L^{-1}).

The high peroxidase activity of WS_2 QDs was studied via steady-state kinetics. The Michaelis–Menten equation was used for obtaining initial rates of each series of reactions (Figures S2a,b, Supplementary Materials):

$$1/\upsilon = (K_m / V_m).(1/[S]) + 1/V_{\max}.$$

Values of K_m and V_{max} were calculated from Lineweaver–Burk graphs (Figure S2c,d, Supplementary Materials) and the results were compared with the amounts of HRP (Table S1, Supplementary Materials). The lower K_m indicates a higher affinity of WS_2 QDs for the substrates (both TMB and H_2O_2). Accordingly, a very low concentration of substrate was needed to reach the maximum performance. The prepared nanoperoxidase had higher efficiency in the TMB–H_2O_2 reaction compared with HRP (Table S1, Supplementary Materials).

2.2.2. Electrochemical Investigations

The activity mechanism of WS_2 QDs in H_2O_2 reactions was also studied via an electrochemical method. A modified glassy carbon electrode (GCE) was prepared, and its electrocatalytic activity in the electrochemical reduction of H_2O_2 was investigated via cyclic voltammetry and amperometry methods (Figure 4). In the presence of H_2O_2, the electrode indicated an obvious current. However, no evident current was observed in the absence of H_2O_2. The data demonstrated that electrons were transferred from electrode to H_2O_2 molecules due to the presence of WS_2 QDs that facilitated electron transfer from the electrode to H_2O_2. As a result, the electron transfer to H_2O_2 could occur with a higher rate, which enhanced the reduction rate of H_2O_2.

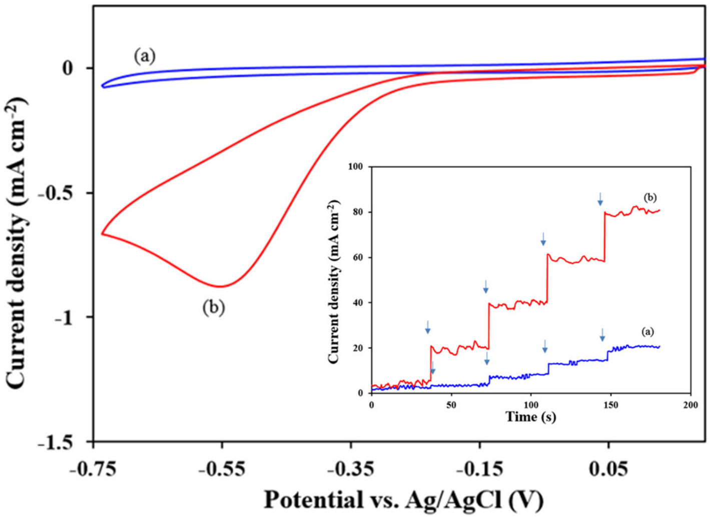

Figure 4. Cyclic voltammograms of (**a**) bare and (**b**) modified glassy carbon electrode (GCE) with WS_2 QDs for 1 mmol·L^{-1} H_2O_2; the inset shows the amperometric response of (**a**) bare GCE and (**b**) modified GCE with WS_2 QDs for successive additions of 10 μmol·L^{-1} H_2O_2.

2.2.3. Chemiluminescence Investigations

As reported in previous literature, H_2O_2 could oxidize RB in an alkaline environment and produce weak CL emission [48]. In this study, it was observed that the presence of WS_2 QDs along with SDS led to a significant increase in CL emission (Table S2, Supplementary Materials). SDS was indicated as facilitating behavior in a similar reaction. Time profiles of CL emission (Figure 5) confirmed the catalytic activity of QDs in the RB–H_2O_2 reaction. On the other hand, the reactants used for the synthesis of the nanomaterials did not show an obvious enhancement effect on the intensity of emission (Table S2, Supplementary Materials). Thus, the observed catalytic activity was attributed to the presence of WS_2 QDs.

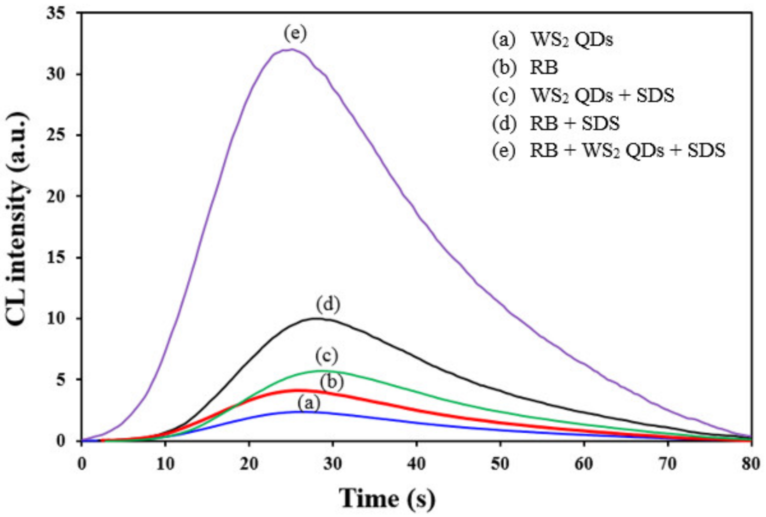

Figure 5. Kinetic profiles of the H_2O_2–RB chemiluminescence (CL) system in the following conditions: (**a**) WS_2 QDs (1 mmol·L^{-1}); (**b**) rhodamine B (RB; 10^{-5} mol·L^{-1}); (**c**) WS_2 QDs (1 mmol·L^{-1}) + SDS (4 × 10^{-3} mol·L^{-1}); (**d**) RB (10^{-5} mol·L^{-1}) + SDS (4 × 10^{-3} mol·L^{-1}); and (**e**) RB (10^{-5} mol·L^{-1}) + WS_2 QDs (1 mmol·L^{-1}) + SDS (4 × 10^{-3} mol·L^{-1}).

2.3. Catalytic Activity of WS_2 QDs

The catalytic effect of used QDs is described below.

QDs catalyze H_2O_2 decomposition and lead to the production of more anionic radicals ($^{\bullet}OH$ and $O_2^{\bullet -}$) and, therefore, improve RB oxidation. The catalytic capacity of WS_2 QDs was discriminated by its incremental effect on CL intensity (Figure 5). The oxidation reaction of RB could generate oxidized RB molecules in an electronically excited state $[^{\bullet}RB^{-}]^{*}_{ox}$ and RB* which could emit at $\lambda \approx 425$ nm [49,50] and $\lambda \approx 575$ nm [51,52], respectively. As reported in previous research [51,52], $[RB]^{*}_{ox}$ is the most oxidized form that is produced in alkaline environment. In addition, there is an efficient energy transfer between RB molecules and the $[RB]^{*}_{ox}$ species via external plasmon process, which generates RB^{*} as emitters [53]. On the other hand, the probable interaction between RB molecules and QDs was studied based on their fluorescence spectra (Figure 6). Because of this interaction, RB molecules could be adsorbed onto the surface of the QDs, causing the quenching of the fluorescence emission of RB.

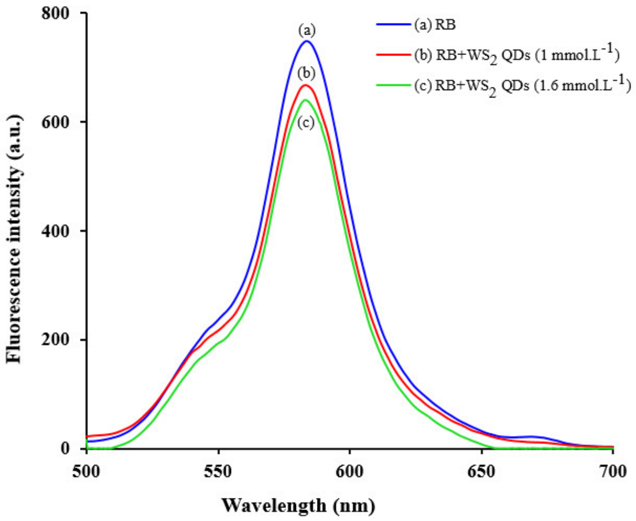

Figure 6. Fluorescence spectra for RB solution (10^{-5} mol·L^{-1}) in the absence (**a**) and presence of 1 mmol·L^{-1} (**b**) or 1.6 mmol·L^{-1} (**c**) WS_2 QDs.

In order to appraise the effect of anionic radicals, some experiments were carried out. Firstly, *tert*-butyl alcohol (as a common •OH radical scavenger) was used to investigate the effect of •OH radicals. In the presence of 0.2 mmol·L^{-1} *tert*-butyl alcohol, about 79% of the CL intensity was attenuated. The result signed that the •OH radicals were highly reactive. Then, to evaluate the role of $O_2^{-\bullet}$ radicals, 0.6 mmol·L^{-1} benzoquinone (as an $O_2^{-\bullet}$ radical scavenger) was added to the system. By adding benzoquinone, just 25% of CL emission was quenched. As a result, $O_2^{-\bullet}$ radicals have a lower effect on the oxidation of RB than •OH radicals.

2.4. Determination of H_2O_2 and Glucose Using a CL System

In order to apply the synthesized peroxidase-like nanomaterials in an analytical scope, they were used for measuring glucose and H_2O_2. In comparison with other investigated techniques, the described stopped-flow CL method had high sensitivity. H_2O_2 and gluconic acid were the products of enzymatic oxidation of glucose by free O_2 and glucose oxidase (GOx). The produced H_2O_2 was injected to the CL flow-cell on WS_2 QDs and SDS-containing RB solution. The currents of the reagents were stopped by switching off the peristaltic pump instantly after observation of first CL emission. Maximum intensity of emitted CL against time was considered as the detection signal.

In order to attain the highest sensitivity, key factors in both the enzymatic process and CL detection step were optimized (Figure S3, Supplementary Materials). As can be seen from the results, the highest signal was obtained via 10 U·mL^{-1} GOx at 6.6 mmol·L^{-1} acetate buffer and 4.4 pH. Also, the optimal amounts in the CL detection process were 10^{-5} mol·L^{-1} RB, 10^{-4} mol·L^{-1} WS_2 QDs, 4 × 10^{-3} mol·L^{-1} SDS, and 10^{-2} mol·L^{-1} NaOH. The higher values of WS_2 QDs caused a decrease in the signal that was attributed to its tendency to react with emitter intermediates.

2.4.1. Analytical Figures of Merit

At optimized conditions, the CL emission intensity of the present system correlated with H_2O_2 concentration (Figure S4a, Supplementary Materials). A calibration graph of emitted CL intensity against concentration of H_2O_2 was obtained in the concentration range of 4–1000 nmol·L^{-1} at optimized conditions (Figure S4b, Supplementary Materials). The detection limit was calculated as 2.4 nmol·L^{-1} for H_2O_2 via $3S_b/m$ (m: slope of calibration graph, and S_b: standard deviation of signals for repetitive determination of five blank solutions) and the linear relationship corresponded to I_{CL} = 866.15C + 34 (R^2 = 0.9999). The previous methods based on nanomaterials for determination of H_2O_2 are provided in Table 1, showing the great features of the described method. Furthermore, the introduced CL system was examined for the determination of glucose. As investigated in Figure S5 (Supplementary Materials), the CL intensity was increased by rising glucose concentration, and a linear calibration graph was obtained using I against C (I and C indicate CL intensity and glucose concentration, respectively). The linear range of the abovementioned method for detection of glucose was 0.01–30 μmol·L^{-1} with a limit of detection (LOD) of 4.2 nmol·L^{-1}, and the linear relationship was obtained as I_{CL} = 10^{11}C + 11465 (R^2 = 0.9996). Good sensitivity with a lower detection limit of the mentioned CL method can be observed from the comparison of its analytical figures with other reported CL systems (Table S3, Supplementary Materials). On the other hand, because of the reliability and rapidity of this method, it is useful for the detection of glucose and other biomolecules.

On the other hand, the precision of the developed method was investigated by computing relative standard deviation (RSD) for the measurement of five similar standard solutions (with a constant concentration of glucose). The RSD values were obtained as 3.15% and 2.69% for 10 and 0.5 μmol·L^{-1} glucose solutions, respectively.

Table 1. An overview on some methods based on nanomaterials for the determination of H_2O_2. L—chemiluminescence; LOD—limit of detection.

Materials Used	Method Applied	LOD (nmol·L^{-1})	Linear Range (µmol·L^{-1})	Stability	Reference
Hemin-functionalized WS_2 nanostructures (NS)	Colorimetric	1500	5–200	-	[54]
Mb nanoporous ZnO films	Electrochemical	2000	10–1800	2 weeks	[55]
WS_2 NS and AgNC mixture	Stopped-flow CL	0.6	0.0025–1.5	1 month	[56]
WS_2 NS/AgNC nanocomposite	Fluorescence	21	0.05–400	1 month	[57]
NiO nanoparticle (NP) modified with 5,10,15,20-tetrakis(4-carboxyl phenyl)-porphyrin	Colorimetric	8000	20–100	2 h	[58]
WS_2 quantum dots (QDs)	Stopped-flow CL	2.4	0.004–1	1 month	This work

In order to study the stability of the prepared sensor, it was used in defined intervals. The results indicated that this sensor could be kept at 4 °C for at least a month, without significant alteration of the CL intensity (Figure S6, Supplementary Materials). Thus, application of this biosensor in public laboratories for medical applications can be useful.

2.4.2. Selectivity

A series of experiments were done to demonstrate the selectivity of the prepared probe for the measurement of constant concentration (1 µmol·L^{-1}) of glucose solutions, in the presence of various concentrations of possible interfering compounds. An endurance limit of 5% was considered for the interfering effect of examined species. The data shown in Figure S7 (Supplementary Materials) indicate that most of studied compounds had no significant interfering effect. Moreover, a 1:500 dilution of real samples was helpful in further elimination of the interfering compound's effects, such that the most of tested species did not show any considerable interfering effect, even at a higher concentration (5 mmol·L^{-1}). Moreover, no important influence was seen in the presence of common cations or anions on the signal of the probe (Table S4, Supplementary Materials). The results confirm the application of the designed biosensor for measuring glucose in real blood samples.

2.4.3. Analysis of Real Samples

The reliability of the improved method was investigated for measuring glucose in human serum samples using a standard addition process (Table S5, Supplementary Materials). For this purpose, the prepared spiked samples were analyzed by adding certain amounts of standard glucose solution to real blood samples. The obtained efficiency demonstrated the reliability of the probe for glucose measuring.

Furthermore, for affirmation of the introduced probe, it was applied for the measurement of a standard reference material (SRM 965 b, frozen serum) (Table S6, Supplementary Materials). The results (4.092 ± 1.435 µmol·L^{-1}) had good compatibility with a certified value (4.194 ± 0.059 µmol·L^{-1}) for SRM which was allotted by the National Institute of Standards and Technology (NIST).

3. Materials and Methods

3.1. Instruments and Materials

GOx, WS_2, HRP, benzoquinone, *tert*-butyl alcohol, and TMB were acquired from Sigma Co. (St. Louis, MO, USA, www.sigmaaldrich.com). Rhodamine B (RB), NaOH, H_2O_2, glucose, and SDS were obtained from Merck Co. (Darmstadt, Germany, www.merck.com). All chemicals were obtained in analytical grade and used without any treatment. All experimental solutions were prepared by double-deionized (DI) water. The morphological assessments of synthesized nanosheets were

carried out with a JEOL HR-TEM (JEM-2200FS, acting at 200 kV, Tokyo, Japan, www.jeol.co.jp). XRD patterns were acquired using a Siemens D5000 X-ray diffractometer (Berlin, Germany, www.siemens.com) with a Cu Kα exciting source (λ = 1.54056 Å). Florescence analyses were performed using an LS-45 PerkinElmer spectrofluorometer (Waltham, MA, USA, www.perkinelmer.com). An S2000 spectrophotometer (WPA Lightwave, Cambridge, England, www.biochrom.co.uk) was used to obtain UV–visible absorption data. An IviumStat potentiostat/galvanostat (Eindhoven, Netherlands, www.ivium.nl) was utilized for electrochemical investigations. CL experiments were done using a Berthold FB12 luminometer (Bad Wildbad, Germany, www.berthold.com).

3.2. Synthesis of WS_2 QDs

For this purpose, 1 g of tungsten sulfide powder was weighed and added to 100 mL of dimethylformamide (DMF). The solution was sonicated for 3 h. The obtained solution was then stirred vigorously in a closed container for 6 h at 140 °C. The yellow solution above the sediment represented the WS_2 QDs, and the sediment was a composite of nanosheets and QDs of tungsten sulfide. In order to eliminate the excessive solvent, the supernatant was evaporated under vacuum at a certain temperature and redispersed in water for further use.

3.3. Investigation of Peroxidase-Like Activity of WS_2 QDs

Colorimetry, electrochemical, and CL techniques were utilized to investigate the peroxidase-like activity of prepared QDs. It should be noted that all of the studies for each method were accomplished in optimized conditions (including pH, buffer type, catalyst, and reagents concentration). Details can be found in the Supplementary Materials.

3.3.1. Colorimetric experiments

Colorimetric experiments were based on the TMB–H_2O_2 reaction. Briefly, 1 mL of WS_2 QDs (1 mmol·L^{-1}) and SDS (4 × 10^{-3} mol·L^{-1}) were added to a 2.5-mL reaction solution containing 2 mmol·L^{-1} TMB and 0.1 mol·L^{-1} acetate buffer (pH 4). Then, 1 mL H_2O_2 solution was added to the mixture and the final volume reached 5 mL with deionized water. After a time period of 10 min, the absorbance of the solution was recorded at 652 nm.

Process for the Investigation of Steady-state Kinetics

The experiments were performed based on the TMB–H_2O_2 reaction using constant conditions. Briefly, a series of solutions containing 0.05 mol.L^{-1} acetate buffer (pH 4) and a certain amount of catalyst (WS_2 QDs (1 mmol.L^{-1})) were prepared. Then, 500 µL of TMB solution (5 mmol·L^{-1}) and different amounts of H_2O_2 (varied from 1 to 200 µmol·L^{-1}) were added. On the other hand, the second series of solutions containing 0.05 mol·L^{-1} acetate buffer (pH 4) and a certain amount of catalyst (WS_2 QDs (1 mmol·L^{-1})) were prepared. Then, a 500-µL H_2O_2 solution (2 mmol·L^{-1}) and different amounts of TMB (varied from 50 to 800 µmol·L^{-1}) were added. The conditions were entirely fixed for all of solutions and their final volumes reached 5 mL using deionized water. Finally, the initial reaction velocities for all solutions were obtained by following their absorbance at 652 nm. In order to obtain Figure S2a,S2b, the velocities were plotted against the varied concentrations of TMB or H_2O_2. Also, the reciprocal velocities against the reciprocal concentrations resulted in Figure S2c,S2d.

3.3.2. Electrochemical Experiments

Electrochemical experiments were performed using modified glassy carbon electrodes (GCE). A dispersion of 3 mg of catalyst (WS_2 QDs and SDS) in 450 µL of DMF and 50 µL of Nafion was mixed and sonicated for 25 min to create a homogeneous ink. On the other hand, GCE electrode was suitably polished and washed by its ultrasonication in ethanol. Then, a small volume of prepared ink (5 µL) was placed on the polished surface of GCE. After evaporation of liquids under

infrared light, the electrode was applied to evaluate the H_2O_2 reduction using cyclic voltammetry (CV) and amperometry techniques.

3.4. Measuring Glucose and H_2O_2 by CL

Glucose measurement: Fifty microliters of standard glucose solution (or prepared real samples) and 50 μL of GOx solution (10 U·mL^{-1}) plus 0.2 mL of acetate buffer (5 mmol·L^{-1}, pH 4.4) were added to a 3-mL Eppendorf tube. In order to complete the enzymatic oxidation of glucose by O_2 and produce H_2O_2, the solution was stored at 37 °C for 5 min. The resulted solution was diluted with distilled water and then used to measure H_2O_2 via luminescence methods as described below.

H_2O_2 detection: The H_2O_2–RB reaction, catalyzed by WS_2 QDs and SDS, involved a flow-mode CL system, used to measure H_2O_2 (Figure 7). The establishment of the CL system was convened by three polytetrafluoroethylene (PTFE) channels (inner diameter of 1.0 mm) to guide the reagents to the CL cell. A 0.01 mol·L^{-1} NaOH solution (in route A) and RB solution (10^{-5} mol·L^{-1}), alone or with WS_2 QDs (1 mmol·L^{-1}), and SDS (4 × 10^{-3} mol·L^{-1}) was traversed along the B path and mixed in a glassy Y-shaped junction and, eventually, kept in a loop with a volume of 150 μL. Distilled water, as a carrier, was transferred via route C through a six-port valve-containing loop and H_2O_2 solution (as an analyte or oxidizing agent) was guided through the D pathway into the CL cell in front of the detector. The carrier current directed the RB solution, which contained WS_2 QDs and SDS, along with NaOH, in the emission region by changing the valve state to the injection mode. After observing the minimum primary CL emission, the reactant flow was immediately stopped by the pump and the amount of emission released over time was recorded. The CL emission resulted from the injection of H_2O_2 solution into the flow cell and the consequent combination of RB in the presence of a catalyst with H_2O_2.

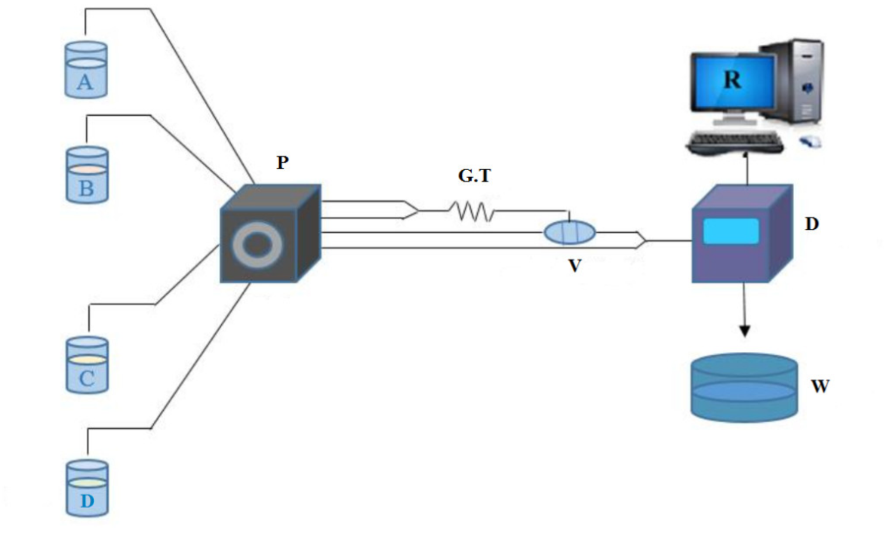

Figure 7. Chemiluminescence flow system applied in this investigation; A: NaOH solution; B: RB solution with or without catalyst; C: carrier; D: H_2O_2 solution; P: peristaltic pump; G.T: glassy triple; V: six-port valve; D: luminometer detector; W: waste; R: personal computer (PC) for processing the obtained data.

3.5. Stability

The stability of the mixed-reagent CL solution (containing RB and peroxidase-like WS_2 QD catalyst) was studied in certain time intervals. The solution was preserved in 4 °C for different times and then was applied for the determination of certain concentrations of glucose. The results confirmed that the mixed reagents can be maintained at 4 °C for at least one month, without any considerable change in CL response (Figure S6). Thus, the offered bioassay can be useful in public laboratories for medical applications.

3.6. Determination of Glucose in Human Blood

For measuring glucose in real samples, collected from healthy volunteers, the samples were centrifuged for 30 min (10,000 rpm). The serums were diluted with phosphate buffer (5 mmol·L^{-1}, pH = 7). Finally, a suitable amount of diluted samples was used to measure glucose as mentioned above.

4. Conclusions

In summary, the mimetic activity of WS_2 QDs was studied via CL, electrochemical, and colorimetry methods. It was found that WS_2 QDs have catalytic activity on the reaction of H_2O_2 reduction. In comparison to HRP, the disadvantages of natural enzymes were rectified. Based on this probe, a sensitive and rapid assay was designed for the determination of H_2O_2 and biomolecules such as blood glucose, using an efficient H_2O_2–RB–WS_2 QD–SDS flow-stopped CL detection system.

Supplementary Materials: The following are available online: Figure S1: XRD pattern of WS_2 QDs; Figure S2: Steady-state kinetic assay and catalytic mechanism of HRP, WS_2 QDs, and the corresponding double-reciprocal plots for catalytic activity of WS_2 QDs and HRP; Figure S3: Effect of (a) RB concentration, (b) NaOH concentration, (c) SDS concentration, and (d) WS_2 QDs concentration on the CL emission intensity of the RB–H_2O_2–NaOH–SDS–WS_2 QD system; Figure S4: (a) CL time profiles of RB–H_2O_2–WS_2 QD–SDS–NaOH system in the presence of different concentrations of H_2O_2 obtained with the stop-flow method, (b) corresponding calibration graph; Figure S5: (a) CL time profiles of the RB–H_2O_2–WS_2 QD–SDS–NaOH system applied for the determination of different concentrations of glucose after its oxidation with GOx (10 U·mL^{-1}) in acetate buffer (pH: 4.4, 5 mmol·L^{-1}); (b) corresponding calibration graph; Figure S6: Catalytic activity of synthesized WS_2 QDs within a specified time after synthesis; Figure S7: Interference of different species on the determination of 5 μmol·L^{-1} glucose; Table S1: Maximum reaction rate (V_m) and Michaelis–Menten constant (K_m) for applied peroxidase-like catalysts compared with HRP; Table S2: Effects of WS_2 QDs and SDS and their procurers on the CL emission of the H_2O_2–RB system; Table S3: A comparison of different analytical techniques for the determination of glucose; Table S4: Interference of different species on the determination of 5 μmol·L^{-1} glucose; Table S5: Glucose detection in human serum samples by designed CL system; Table S6: Glucose detection in standard sample by designed CL system.

Author Contributions: M.H.I. performed the experiments and wrote the paper; A.K. acted as a supervisor; J.H. gave scientific suggestions on this work and edited the paper; M.H. and J.H. analyzed the data; Y.O. edited the paper.

Acknowledgements: We thank the University of Tabriz for the provided support.

Conflicts of Interest: The authors declare no conflicts of interest.

References

1. Salimi, A.; Hallaj, R.; Soltanian, S.; Mamkhezri, H. Nanomolar detection of hydrogen peroxide on glassy carbon electrode modified with electrodeposited cobalt oxide nanoparticles. *Anal. Chim. Acta.* **2007**, *594*, 24–31.
2. Zhang, L.-S.; Wong, G.T. Optimal conditions and sample storage for the determination of H_2O_2 in marine waters by the scopoletin–horseradish peroxidase fluorometric method. *Talanta* **1999**, *48*, 1031–1038.
3. Sang, Y.; Zhang, L.; Li, Y.F.; Chen, L.Q.; Xu, J.L.; Huang, C.Z. A visual detection of hydrogen peroxide on the basis of Fenton reaction with gold nanoparticles. *Anal. Chim. Acta.* **2010**, *659*, 224–228.
4. Shang, L.; Chen, H.; Deng, L.; Dong, S. Enhanced resonance light scattering based on biocatalytic growth of gold nanoparticles for biosensors design. *Biosens. Bioelectron.* **2008**, *23*, 1180–1184.
5. Huang, T.; Garceau, M.E.; Gao, P. Liquid chromatographic determination of residual hydrogen peroxide in pharmaceutical excipients using platinum and wired enzyme electrodes. *J. Pharmaceut. Biomed.* **2003**, *31*, 1203–1210.
6. Zhu, M.; Han, S.; Yuan, Z. β-Cyclodextrin polymer as the immobilization matrix for peroxidase and mediator in the fabrication of a sensor for hydrogen peroxide. *J. Electroanal. Chem.* **2000**, *480*, 255–261.
7. Xu, Y.; Pehrsson, P.E.; Chen, L.; Zhang, R.; Zhao, W. Double-stranded DNA single-walled carbon nanotube hybrids for optical hydrogen peroxide and glucose sensing. *The J. Phys. Chem. C.* **2007**, *111*, 8638–8643.

8. Dodeigne, C.; Thunus, L.; Lejeune, R. Chemiluminescence as diagnostic tool. A review. *Talanta* **2000**, *51*, 415–439.
9. Khataee, A.; Lotfi, R.; Hasanzadeh, A.; Iranifam, M.; Joo, S.W. Flow-injection chemiluminescence analysis for sensitive determination of atenolol using cadmium sulfide quantum dots. *Spectrochim. Acta. A.* **2016**, *157*, 88–95.
10. Trojanowicz, M.; Kołacińska, K. Recent advances in flow injection analysis. *Analyst* **2016**, *141*, 2085–2139.
11. Su, Y.; Deng, D.; Zhang, L.; Song, H.; Lv, Y. Strategies in liquid-phase chemiluminescence and their applications in bioassay. *TrAC-Trend. Anal. Chem.* **2016**, *82*, 394–411.
12. Pokropivny, V.; Ivanovskii, A.L. New nanoforms of carbon and boron nitride. *Russ. Chem. Rev* **2008**, *77*, 837.
13. Kim, Y.T.; Han, J.H.; Hong, B.H.; Kwon, Y.U. Electrochemical synthesis of CdSe quantum-dot arrays on a graphene basal plane using mesoporous silica thin-film templates. *Adv. Mater.* **2010,** *22*, 515–518.
14. Zhang, G.; Wang, D. Colloidal lithography-the art of nanochemical patterning. *Chem. Asian, J.* **2009**, *4*, 236–245.
15. Gautam, U.K.; Vivekchand, S.; Govindaraj, A.; Rao, C. GaS and GaSe nanowalls and their transformation to Ga_2O_3 and GaN nanowalls. *Chem. Commun.* **2005**, 3995–3997.
16. Lee, J.Y.; Hong, B.H.; Kim, W.Y.; Min, S.K.; Kim, Y.; Jouravlev, M.V.; Bose, R.; Kim, K.S.; Hwang, I.-C.; Kaufman, L.J. Near-field focusing and magnification through self-assembled nanoscale spherical lenses. *Nature* **2009**, *460*, 498.
17. Stouwdam, J.W.; Janssen, R.A. Red, green, and blue quantum dot LEDs with solution processable ZnO nanocrystal electron injection layers. *J. Mater. Chem.* **2008**, *18*, 1889–1894.
18. Lee, W.; Kang, S.H.; Kim, J.-Y.; Kolekar, G.B.; Sung, Y.-E.; Han, S.-H. TiO_2 nanotubes with a ZnO thin energy barrier for improved current efficiency of CdSe quantum-dot-sensitized solar cells. *Nanotechnology* **2009**, *20*, 335706.
19. Mokerov, V.; Fedorov, Y.V.; Velikovski, L.; Scherbakova, M.Y. New quantum dot transistor. *Nanotechnology* **2001**, *12*, 552.
20. Ustinov, V.; Zhukov, A.; Kovsh, A.; Mikhrin, S.; Maleev, N.; Volovik, B.; Musikhin, Y.G.; Shernyakov, Y.M.; Kondat'eva, E.Y.; Maximov, M. Long-wavelength quantum dot lasers on GaAs substrates. *Nanotechnology* **2000**, *11*, 397.
21. Bagher, A.M. Quantum dots applications. *Sensors & Transducers* **2016**, *198*, 37.
22. Kim, H.; Beack, S.; Han, S.; Shin, M.; Lee, T.; Park, Y.; Kim, K.S.; Yetisen, A.K.; Yun, S.H.; Kwon, W. Multifunctional photonic nanomaterials for diagnostic, therapeutic, and theranostic applications. *Adv. Mater.* **2018**, *30*, 1701460.
23. Kvaratskhelia, M.; Winkel, C.; Thorneley, R.N. Purification and characterization of a novel class III peroxidase isoenzyme from tea leaves. *Plant. Physiol.* **1997**, *114*, 1237–1245.
24. Wei, H.; Wang, E. Nanomaterials with enzyme-like characteristics (nanozymes): Next-generation artificial enzymes. *Chem. Soc. Rev.* **2013**, *42*, 6060–6093.
25. Wiester, M.J.; Ulmann, P.A.; Mirkin, C.A. Enzyme mimics based upon supramolecular coordination chemistry. *Angew. Chem. Int. Edit.* **2011**, *50*, 114–137.
26. Gao, L.; Wu, J.; Lyle, S.; Zehr, K.; Cao, L.; Gao, D. Magnetite nanoparticle-linked immunosorbent assay. *J. Phys. Chem. C.* **2008**, *112*, 17357–17361.
27. Lin, T.; Zhong, L.; Guo, L.; Fu, F.; Chen, G. Seeing diabetes: Visual detection of glucose based on the intrinsic peroxidase-like activity of MoS_2 nanosheets. *Nanoscale* **2014**, *6*, 11856–11862.
28. Mu, J.; Wang, Y.; Zhao, M.; Zhang, L. Intrinsic peroxidase-like activity and catalase-like activity of Co_3O_4 nanoparticles. *Chem. Commun.* **2012**, *48*, 2540–2542.
29. André, R.; Natálio, F.; Humanes, M.; Leppin, J.; Heinze, K.; Wever, R.; Schröder, H.C.; Müller, W.E.; Tremel, W. V_2O_5 nanowires with an intrinsic peroxidase-like activity. *Adv. Funct. Mater.* **2011**, *21*, 501–509.
30. Song, Y.; Wang, X.; Zhao, C.; Qu, K.; Ren, J.; Qu, X. Label-free colorimetric detection of single nucleotide polymorphism by using single-walled carbon nanotube intrinsic peroxidase-like activity. *Chem. Eur. J.* **2010**, *16*, 3617–3621.
31. Song, Y.; Qu, K.; Zhao, C.; Ren, J.; Qu, X. Graphene oxide: Intrinsic peroxidase catalytic activity and its application to glucose detection. *Adv. Mater.* **2010**, *22*, 2206–2210.
32. Shi, W.; Wang, Q.; Long, Y.; Cheng, Z.; Chen, S.; Zheng, H.; Huang, Y. Carbon nanodots as peroxidase mimetics and their applications to glucose detection. *Chem. Commun.* **2011**, *47*, 6695–6697.

33. Tenne, R. Inorganic nanotubes and fullerene-like nanoparticles. *J. Mater. Res.* **2006**, *21*, 2726–2743.
34. Xu, G.; Zeng, S.; Zhang, B.; Swihart, M.T.; Yong, K.-T.; Prasad, P.N. New generation cadmium-free quantum dots for biophotonics and nanomedicine. *Chem. Rev.* **2016**, *116*, 12234–12327.
35. Tenne, R.; Margulis, L.; Genut, M; Hodes, G. Polyhedral and cylindrical structures of tungsten disulphide. *Nature* **1992**, *360*, 444–446.
36. Khataee, A.; Eghbali, P.; Irani-nezhad, M.H.; Hassani, A. Sonochemical synthesis of WS_2 nanosheets and its application in sonocatalytic removal of organic dyes from water solution. *Ultrason. Sonochem.* **2018**, *48*, 329–339.
37. Xu, S.; Li, D.; Wu, P. One-pot, facile, and versatile synthesis of monolayer MoS_2/WS_2 quantum dots as bioimaging probes and efficient electrocatalysts for hydrogen evolution reaction. *Adv. Funct. Mater.* **2015**, *25*, 1127–1136.
38. Zhang, X.; Lai, Z.; Liu, Z.; Tan, C.; Huang, Y.; Li, B.; Zhao, M.; Xie, L.; Huang, W.; Zhang, H. A facile and universal top-down method for preparation of monodisperse transition-metal dichalcogenide nanodots. *Angew. Chem. Int. Edit.* **2015**, *54*, 5425–5428.
39. Kim, M.-J.; Jeon, S.-J.; Kang, T.W.; Ju, J.-M.; Yim, D.; Kim, H.-I.; Park, J.H.; Kim, J.-H. 2H-WS_2 quantum dots produced by modulating the dimension and phase of 1T-nanosheets for antibody-free optical sensing of neurotransmitters. *ACS. Appl. Mater. Interfaces.* **2017**, *9*, 12316–12323.
40. Das, S.; Kim, M.; Lee, J.-W.; Choi, W. Synthesis, properties, and applications of 2-D materials: A comprehensive review. *Crit. Rev. Solid State* **2014**, *39*, 231–252.
41. Štengl, V.; Henych, J.; Slušná, M.; Ecorchard, P. Ultrasound exfoliation of inorganic analogues of graphene. *Nanoscale. Res. Lett.* **2014**, *9*, 167.
42. Gopalakrishnan, D.; Damien, D.; Shaijumon, M.M. MoS_2 quantum dot-interspersed exfoliated MoS_2 nanosheets. *ACS Nano* **2014**, *8*, 5297–5303.
43. Yang, Z.-C.; Wang, M.; Yong, A.M.; Wong, S.Y.; Zhang, X.-H.; Tan, H.; Chang, A.Y.; Li, X.; Wang, J. Intrinsically fluorescent carbon dots with tunable emission derived from hydrothermal treatment of glucose in the presence of monopotassium phosphate. *Chem. Commun.* **2011**, *47*, 11615–11617.
44. Yang, Y.; Cui, J.; Zheng, M.; Hu, C.; Tan, S.; Xiao, Y.; Yang, Q.; Liu, Y. One-step synthesis of amino-functionalized fluorescent carbon nanoparticles by hydrothermal carbonization of chitosan. *Chem. Commun.* **2012**, *48*, 380–382.
45. Ren, X.; Pang, L.; Zhang, Y.; Ren, X.; Fan, H.; Liu, S.F. One-step hydrothermal synthesis of monolayer MoS_2 quantum dots for highly efficient electrocatalytic hydrogen evolution. *J. Mater. Chem. A.* **2015**, *3*, 10693–10697.
46. Lin, L.; Xu, Y.; Zhang, S.; Ross, I.M.; Ong, A.C.; Allwood, D.A. Fabrication of luminescent monolayered tungsten dichalcogenides quantum dots with giant spin-valley coupling. *ACS Nano* **2013**, *7*, 8214–8223.
47. Yan, Y.; Zhang, C.; Gu, W.; Ding, C.; Li, X.; Xian, Y. Facile synthesis of water-soluble WS_2 quantum dots for turn-on fluorescent measurement of lipoic acid. *J. Phys. Chem. C* **2016**, *120*, 12170–12177.
48. Han, S.; Liu, E.; Li, H. Flow injection chemiluminescence determination of hemin using the rhodamine B–H_2O_2–NaOH system. *Microchim. Acta* **2005**, *149*, 281–286.
49. Ma, Y.; Zhou, M.; Jin, X.; Zhang, B.; Chen, H.; Guo, N. Flow–injection chemiluminescence determination of ascorbic acid by use of the cerium (IV)–Rhodamine B system. *Anal. Chim. Acta* **2002**, *464*, 289–293.
50. Ma, Y.; Jin, X.; Zhou, M.; Zhang, Z.; Teng, X.; Chen, H. Chemiluminescence behavior based on oxidation reaction of rhodamine B with cerium (IV) in sulfuric acid medium. *Anal. Chim. Acta* **2003**, *489*, 173–181.
51. Hassanzadeh, J.; Khataee, A.; Oskoei, Y.M.; Fattahi, H.; Bagheri, N. Selective chemiluminescence method for the determination of trinitrotoluene based on molecularly imprinted polymer-capped ZnO quantum dots. *New J. Chem.* **2017**, *41*, 10659–10667.
52. Vahid, B.; Hassanzadeh, J.; Abolhasani, J.; Khodakarami, B. Inhibition of rhodamine B–ferricyanide chemiluminescence by Au nanoparticles toward the sensitive determination of mercury (II) ions. *Microchim. Acta* **2016**, *126*, 326–331.
53. Hassanzadeh, J.; Khataee, A. Ultrasensitive chemiluminescent biosensor for the detection of cholesterol based on synergetic peroxidase-like activity of MoS_2 and graphene quantum dots. *Talanta* **2018**, *178*, 992–1000.
54. Chen, Q.; Chen, J.; Gao, C.; Zhang, M.; Chen, J.; Qiu, H. Hemin-functionalized WS_2 nanosheets as highly active peroxidase mimetics for label-free colorimetric detection of H_2O_2 and glucose. *Analyst* **2015,** *140*, 2857–2863.

55. Topoglidis, E.; Astuti, Y.; Duriaux, F.; Grätzel, M.; Durrant, J.R. Direct electrochemistry and nitric oxide interaction of heme proteins adsorbed on nanocrystalline tin oxide electrodes. *Langmuir* **2003**, *19*, 6894–6900.
56. Khataee, A.; Irani-Nezhad, M.H.; Hassanzadeh, J. Improved peroxidase mimetic activity of a mixture of WS_2 nanosheets and silver nanoclusters for chemiluminescent quantification of H_2O_2 and glucose. *Microchim. Acta.* **2018**, *185*, 190.
57. Khataee, A.; Irani-nezhad, M.H.; Hassanzadeh, J.; Joo, S.W. Superior peroxidase mimetic activity of tungsten disulfide nanosheets/silver nanoclusters composite: Colorimetric, fluorometric and electrochemical studies. *J. Colloid. Interf. Sci.* **2018**, *515*, 39–49.
58. Liu, Q.; Yang, Y.; Li, H.; Zhu, R.; Shao, Q.; Yang, S.; Xu, J. NiO nanoparticles modified with 5, 10, 15, 20-tetrakis (4-carboxyl pheyl)-porphyrin: Promising peroxidase mimetics for H_2O_2 and glucose detection. *Biosens. Bioelectron.* **2015**, *64*, 147–153.

Sample Availability: Samples of the compounds are available from the authors

© 2019 by the authors. Licensee MDPI, Basel, Switzerland. This article is an open access article distributed under the terms and conditions of the Creative Commons Attribution (CC BY) license (http://creativecommons.org/licenses/by/4.0/).
